# Supplementary material for: Identification of photoperiod-induced specific miRNAs in the adrenal glands of Sunite sheep (Ovis aries)
Source: Front Vet Sci. 2022 Jul 22;9:888207. doi: 10.3389/fvets.2022.888207 (PMC9354845; doi:10.3389/fvets.2022.888207)
Supplement: Supplementary file 1 [file Data_Sheet_1.ZIP › Supplementary file/miRDeep2 file.zip!/miRDeep2 file/Know_Structure/SP42A2/oar-mir-10b.pdf]

miRBase precursor : oar-mir-10b
Total read count : 873257
oar-miR-10b read count : 869593
remaining reads : 3664

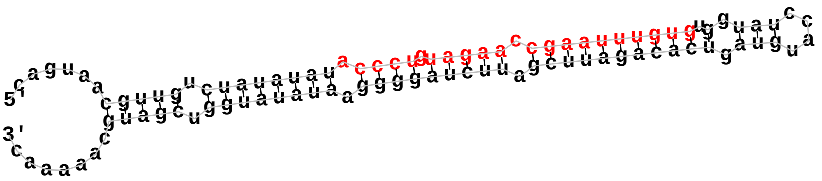

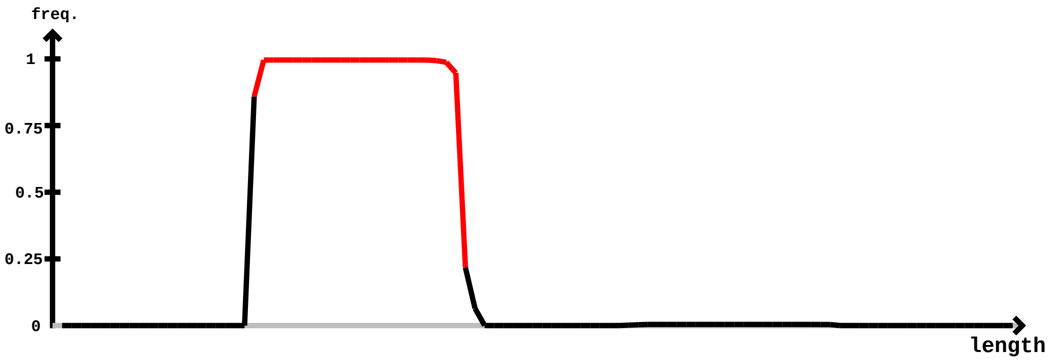

oar-miR-10b

```
5'- caguaacguugucuauauauacccuguagaaccgaauuugugugguauccauguagucacagauucgauucuaggggaauauauggucgaugcaaaaac   -3'   exp
    ......(((((.((((((((.((((.(((((.((((((((((.(.(((....))).))))))))))).))))))))).)))))))).)))))....... reads   mm   sample
    caguaacguugucuauaua................................................................................ 2   0   seq
    ..................auacccuguagaaccgaauuu............................................................ 1   0   seq
    ..................auacccuguagaaccgaauuugu.......................................................... 17   0   seq
    ..................auacccuguagaaccgaauuugug......................................................... 2   0   seq
    ...................uacccuguagaaccgaa............................................................... 23   0   seq
    ...................uacccuguagaaccgaau.............................................................. 150   0   seq
    ...................uacccuguagaaccgaauu............................................................. 1831   0   seq
    ...................uacccuguagaaccgaauuu............................................................ 4706   0   seq
    ...................uacccuguagaaccgaauuug........................................................... 31773   0   seq
    ...................uacccuguagaaccgaauuugu.......................................................... 594679   0   seq
    ...................uacccuguagaaccgaauuugug......................................................... 94780   0   seq
    ...................uacccuguagaaccgaauuugugu........................................................ 21976   0   seq
    ...................uacccuguagaaccgaauuugugug....................................................... 33   0   seq
    ...................uacccuguagaaccgaauuuguguggu..................................................... 1   0   seq
    ...................uacccuguagaaccgaauuuguguggu..................................................... 2   0   seq
    ...................uacccuguagaaccgaauuuguguggua.................................................... 1   0   seq
    ....................acccuguagaaccgaau.............................................................. 10   0   seq
    ....................acccuguagaaccgaauu............................................................. 163   0   seq
    ....................acccuguagaaccgaauuu............................................................ 585   0   seq
    ....................acccuguagaaccgaauuug........................................................... 2963   0   seq
    ....................acccuguagaaccgaauuugu.......................................................... 42626   0   seq
    ....................acccuguagaaccgaauuugug......................................................... 38977   0   seq
    ....................acccuguagaaccgaauuugugu........................................................ 34060   0   seq
    ....................acccuguagaaccgaauuugugug....................................................... 123   0   seq
    ....................acccuguagaaccgaauuuguguggg..................................................... 10   0   seq
    ....................acccuguagaaccgaauuuguguggu..................................................... 1   0   seq
    .....................cccuguagaaccgaauuu............................................................ 1   0   seq
    .....................cccuguagaaccgaauuug........................................................... 3   0   seq
    .....................cccuguagaaccgaauuugu.......................................................... 15   0   seq
    .....................cccuguagaaccgaauuugug......................................................... 13   0   seq
    .....................cccuguagaaccgaauuugugu........................................................ 33   0   seq
    ......................ccuguagaaccgaauuug........................................................... 2   0   seq
    ......................ccuguagaaccgaauuugu.......................................................... 10   0   seq
    ......................ccuguagaaccgaauuugug......................................................... 5   0   seq
    ......................ccuguagaaccgaauuugugu........................................................ 8   0   seq
```

oar-miR-10b

caguaacguugucuauauauacccuguagaaccgaauuugugugguauccauguagucacagauucgauucuaggggaauauauggucgaugcaaaaac			
.......................cuguagaaccgaauuugu..	4	0	seq
.......................cuguagaaccgaauuugug...	2	0	seq
.......................cuguagaaccgaauuugugu..	1	0	seq
........................uguagaaccgaauuugu..	1	0	seq
........................uguagaaccgaauuugug...	1	0	seq
........................uguagaaccgaauuugugu..	1	0	seq
....ucacagauucgauucuaggggaau...................	2	0	seq
....cacagauucgauucuagggga.....................	4	0	seq
....cacagauucgauucuaggggaa....................	4	0	seq
....cacagauucgauucuaggggaau...................	12	0	seq
....acagauucgauucuagggg......................	3	0	seq
....acagauucgauucuagggga.....................	51	0	seq
....acagauucgauucuaggggaa....................	68	0	seq
....acagauucgauucuaggggaau...................	1136	0	seq
....acagauucgauucuaggggaaua..................	4	0	seq
....cagauucgauucuagggg......................	4	0	seq
....cagauucgauucuagggga.....................	53	0	seq
....cagauucgauucuaggggaa....................	40	0	seq
....cagauucgauucuaggggaau...................	1318	0	seq
....cagauucgauucuaggggaaua..................	21	0	seq
....cagauucgauucuaggggaauau.................	9	0	seq
....agauucgauucuagggg......................	1	0	seq
....agauucgauucuagggga.....................	10	0	seq
....agauucgauucuaggggaa....................	9	0	seq
....agauucgauucuaggggaau...................	833	0	seq
....agauucgauucuaggggaaua..................	42	0	seq
....agauucgauucuaggggaauau.................	35	0	seq
....agauucgauucuaggggaauaua................	2	0	seq
....gauucgauucuaggggaaua..................	1	0	seq
